# Extranodal natural killer/T-cell lymphoma invading a patient’s heart: A rare case report and literature review

**DOI:** 10.3389/fonc.2022.1011894

**Published:** 2022-12-07

**Authors:** Yifei Xiang, Xueqin Lin, Xiaoling Cai, Shuang Qu, Kai-Yang Lin

**Affiliations:** ^1^ Department of Cardiology, Fujian Provincial Hospital, Shengli Clinical Medical College of Fujian Medical University, Fuzhou, China; ^2^ Fujian Provincial Key Laboratory of Cardiovascular Disease, Fujian Cardiovascular Institute, Fujian Provincial Center for Geriatrics, Fujian Clinical Medical Research Center for Cardiovascular Diseases, Fuzhou, China; ^3^ Department of Hematology, Fujian Provincial Hospital, Shengli Clinical Medical College of Fujian Medical University, Fuzhou, China; ^4^ Department of Hematology, Fujian Heart Failure Center Alliance, Fuzhou, China

**Keywords:** extranodal natural killer/T-cell lymphoma, heart failure, poor prognosis, targeted therapy, PET-CT

## Abstract

Extranodal NK/T-cell lymphoma (ENKTL) is a rare but aggressive subtype of non-Hodgkin lymphoma, which is derived from NK cells or T cells. There are very few cases of ENKTL invading the heart. Only 12 cases of ENKTL invading the heart have been reported in the English literature. Due to the rarity of this lymphoma, an effective therapeutic strategy has not been defined. Here, we present a case of a 51-year-old Chinese male with extranodal NK/T-cell lymphoma invading the heart and review the literature. The patient received a chemotherapy regimen of PD1 monoclonal antibody (Sintilimab) in combination with first-line P-Gemox. The patient survived for 2 months after diagnosis.

## Introduction

Extranodal NK/T-cell lymphoma is a rare non-Hodgkin lymphoma with dismal outcomes and limited treatment options. According to anatomic origin and clinical manifestations, ENKTL can be divided into nasal, non-nasal, and aggressive/leukemia subtypes. Of the three types, the non-nasal type has much lower survival rate (1). The incidence of this disease is higher in China than in Western countries, and the prognosis is poor with traditional treatments (2, 3). Improving the treatment is an urgent requirement.

Here we report a patient having ENKTL invading the heart. Initially, the patient’s heart failure was thought to be due to pre-existing hypertrophic obstructive cardiomyopathy. Later, with the elevation of cardiac protein, rapid progression of heart failure and MODS, the patient was considered to have acute myocarditis, and finally ENKTL invading the heart was considered by biopsy of nodules found in the patient’s body and whole-body PET-CT.

## Case persentation

A 51-year-old man was admitted to our hospital with weakness and recurrent shortness of breath for almost a year, which was aggravated for two days. Two months prior to admission, a echocardiography showed left ventricular ejection fraction (LVEF) = 57% (**Table 1**). He was treated with alcohol ablation for hypertrophic obstructive cardiomyopathy 6 years ago. Laboratory tests after admission showed N-terminal pro-brain natriuretic peptide(NT-proBNP): 9077.00Pg/ml, Troponin T: 0.32ng/ml, ALT: 274U/L, AST: 432U/L, D dimer: 6.30mg/L. Therefore, acute heart failure is considered based on the patient’s condition and laboratory tests. The patient’s symptoms suddenly deteriorated two days after admission without any obvious cause, and laboratory tests found ALT: 1997U/L, AST: 2552U/L, D dimer:>35mg/L, LDH:2900U/L. The patient presenting acute heart failure combined with acute liver failure, MODS can be diagnosed. Thus, the patient was transferred to the ICU for further treatment.

**Table 1:** Summary of patient echocardiographic changes.

Time of Echocardiography	LVEF	LVOTG
November 2015 (Pre-Alcohol Ablation)	64.8%	125mmHg
December 2015 (Post alcohol ablation)	64.4%	40mmHg
March 2018 (Post alcohol ablation review)	70.8%	26mmHg
June 2021 (Two months prior to admission)	57%	6.4mmHg
August 2021 (Transferred to ICU)	38%	4mmHg
September 2021 (Transferred to Hematology department)	38%	3.6mmHg

**Figure 1:**
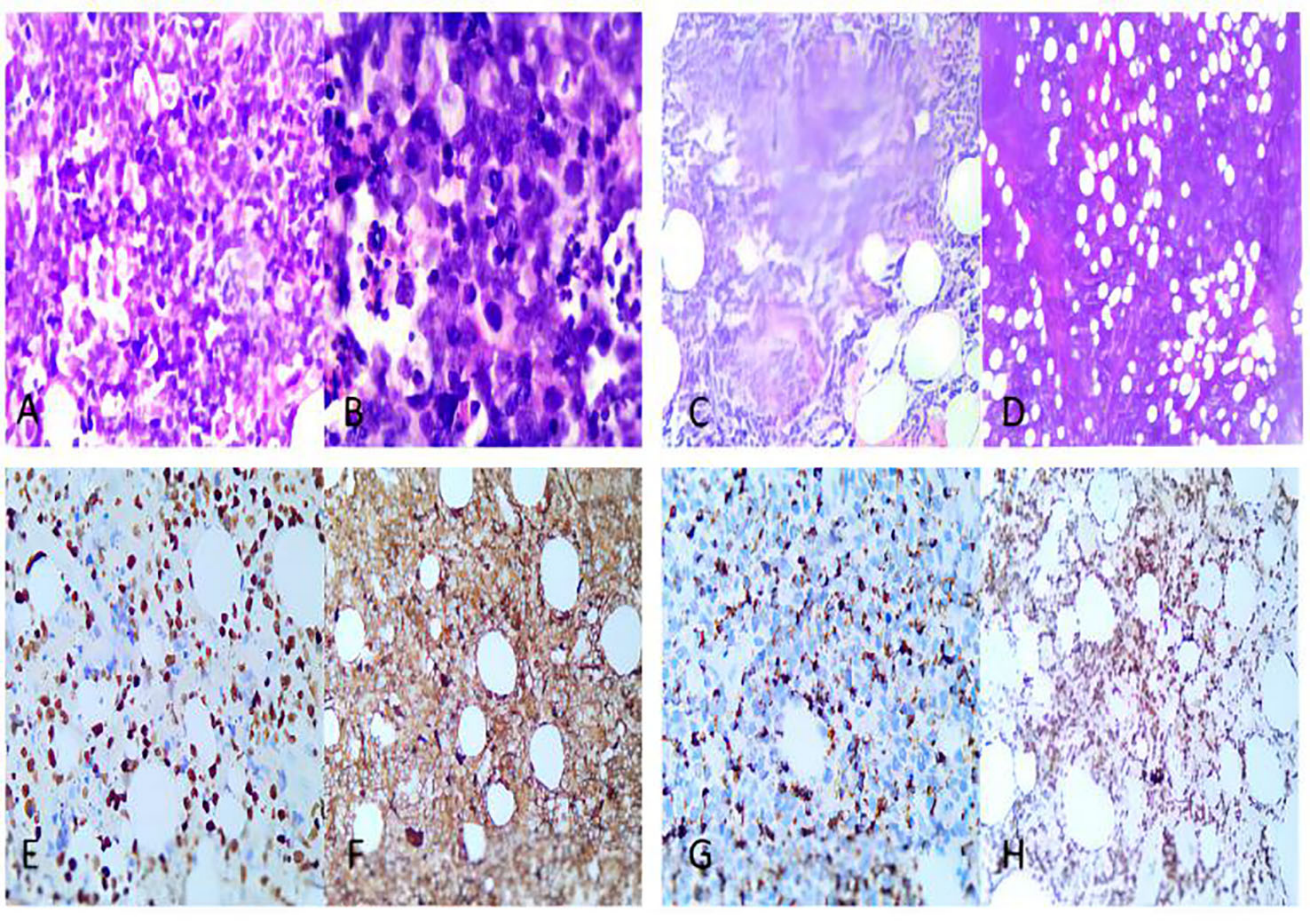
Pathological and immunohistochemical images of the patient. **(A, B)**. Obviously heterogeneous medium-sized tumor cells with apoptotic nuclear fragmentation. **(C)**. Tumor cells angiodestructive growth **(D)**. Subcutaneous interfatty infiltration with extensive necrosis. **(E)**. EBER positive. **(F)**. CD3-positive. **(G)**. TIA1-positive. **(H)**. Ki-67 positive, shows high value-added index.

**Figure 2:**
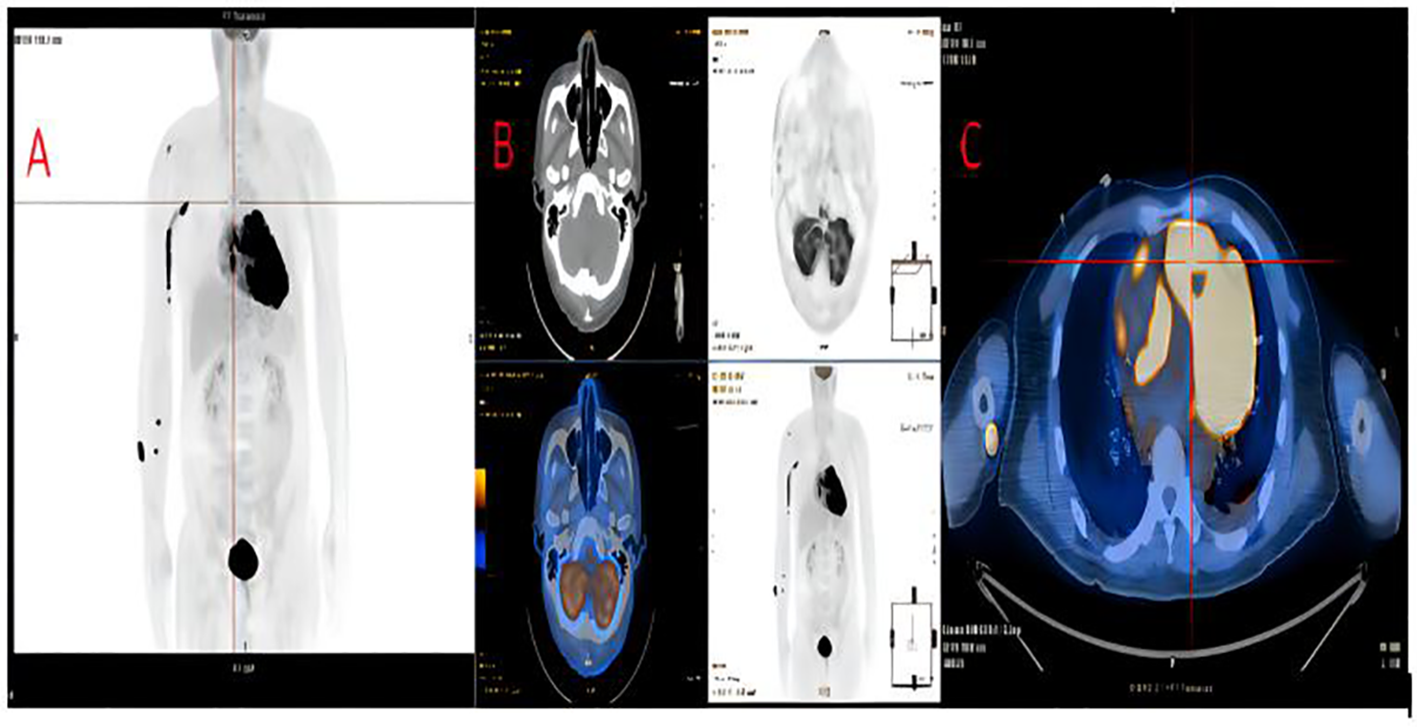
The whole pet-ct of this patient. **(A)**. Multiple involvement throughout the body, including the heart, blood vessels, muscles, and skin. **(B)**. No involvement of the nasal cavity. **(C)**. Cardiac involvement: including the LV as well as the funnel of the RV.

After transferred to ICU, the patient’s symptoms further deteriorate with oliguria and significantly increased NT-proBNP. Acute heart failure combined with acute kidney injury was taken into account. While in the ICU, a review of laboratory indicators indicated a rapid progression of NT-proBNP(25950Pg/ml), a sharp rise in troponin T(3.29ng/ml), and a progressive rise in ALT(2017U/L) and AST(2552U/L). By this time, the patient’s pre-existing hypertrophic obstructive cardiomyopathy could no longer explain the patient’s deteriorating condition. Consequently, fulminant myocarditis was taken into consideration. After administration of high dose methylprednisolone, the patient’s symptoms were relieved. Echocardiography revealed significant thickening of the septum and left ventricular wall, pericardial effusion with decreased left ventricular myocardial contractility(LVEF=38%) (**Table 1**). The patient was treated with pericardiocentesis to drain the pericardial fluid and sent it for examination to determine its nature. And the nature of the pericardial fluid returned showed lymphoma cells. Meanwhile, because of the progression of an inadvertently discovered groin swelling, excision of the inguinal mass was performed, post-operative pathological return: moderate-sized lymphoid tumor cells showing diffuse infiltration with multifocal map-like necrosis. Immunohistochemistry showed: Ki-67(70%+), CD20 (–), CD79a(Focal weak +), PAX5(-), CD3(++), CD5(++), CD43(++), CD4(-), CD8(++), CD56(-), TIA-1 (++), EBER(+), CD30(-), ALKp80(-), TdT(-), CD123(-), CD68(-), CD10(-), BCL6(-), MUMI(++). Combined with the above results, the diagnosis of extranodal NK/T-cell lymphoma(ENKTL) was confirmed (**Figure 1**). Furthermore, PET-CT of the whole body demonstrated that multiple involvement of the heart, skin, blood vessels and muscles without nasal involvement (**Figure 2**). Hence, the patient was regarded as having ENKTL invading the heart. The patient’s rapid progression of heart failure, myocardial injury and MODS were taken into account to be caused by the progressive myocardial infiltration of ENKTL. The palliative effect of glucocorticoids on the patient was considered to be due to the inhibition of the cytokine storm caused by ENKTL rather than inhibition of myocardial inflammation. The reason for this is that the effect of glucocorticosteroids on the patient is rapidly diminishing and the patient’s cardiac function is not improving significantly.

After the patient’s condition stabilized and all laboratory indicators improved, he was transferred to the hematology department for chemotherapy. The results of echocardiography were similar to the previous results.(LVEF=38%) and laboratory tests showed NT-proBNP: 10246.00Pg/ml, Troponin T: 0.84ng/ml. Considering the severity of the patient’s disease, first-line P-Gemox regimen combined with PD-1 monoclonal antibody was administered. This regimen included sintilimab(200mg, day0), gemcitabine(1.8g, day1, day8), oxaliplatin(230mg, day1) and pegaspargase(3750U, day1). Taking into account the poor prognosis of ENKTL invasion of the heart combined with the patient’s physical condition at the time, the dose used in this patient’s chemotherapy regimen was adequate. During chemotherapy the patient’s nodules did not improve, and chidamide(30mg, BIW) was given to improve the efficacy of the treatment. At the end of the current chemotherapy course, the patient’s nodules did not improve and even new nodules appeared. In the meantime, the patient’s heart function also did not show improvement, and laboratory results showed NT-proBNP: 10648.00Pg/ml, Troponin T: 0.25ng/ml. Finally, the patients decided to be discharged automatically. After automatic discharge, the patient’s cardiac function deteriorated further, with symptoms of orthopnea, nocturnal paroxysmal dyspnea and edema of both lower limbs. Moreover, the patient’s skin nodules were larger and more numerous than previously, and they were breaking down. In the end, the patient died at home on October 4, 2021.

## Discussion

ENKTL is a kind of aggressive Epstein‐Barr virus (EBV) infection‐related NHL stemmed from mature NK cells and NK‐Like T Cells, and shows highly‐aggressive progression. Nevertheless, to our knowledge, there are only few cases of ENKTL invading the heart, and the non-nasal type accounts for the majority of these cases. The non-nasal ENKTL, which originates in rare sites like the eyes and skin, are more likely to invade the heart than the nasal type, which may partly account for its poor prognosis.

Not only is the number of cases rare, but patients with ENKTL invading the heart are also prone to misdiagnosis of myocarditis, heart failure and arrhythmias. Whole-body PET-CT with echocardiography is indispensable in the diagnostic process of such diseases. Moreover, this type of patient has poor prognosis, often with death as the clinical outcome (**Table 2**) (1, 4–14).

**Table 2:** Reported cases of ENKTL invading the heart and our case (1-12).

Case source	Type	Race	Age/Sex	Cardiac manifestations	Results of chemotherapy	Clinical outcome
1	Non-nasal	Yellow	51/M	Heart failure	No improvement	Died
2. Asian Cardiovasc Thorac Ann. 2019. 27(3): 210-212.	Non-nasal	Yellow	38/M	Arrhythmia	Unknown	Unknown
3. Intern Med.2014. 53(20):2333-6.	Non-nasal	Yellow	23/M	Arrhythmia	Death due to cardiac arrhythmia during chemotherapy	Died
4. J Clin Oncol.2011.29(34): e833-6.	Non-nasal	Yellow	25/M	Myocarditis	Died without chemotherapy	Died
5.Hematol Rep.2011. 3(2): e9.	Non-nasal	White	54/M	Cardiac mass	No improvement after palliative chemotherapy	Died
6. 6. BMJ Case Rep. 2020.13(1).	Non-nasal	White	18/M	Myocarditis	Improvement	Alive
7. Acta Oncol. 2009.48(4):637-9	Nasal	Yellow	65/M	Arrhythmia	Improvement	Alive
8. ArqBras Oftalmol. 2012.75(6):430-2.	Non-nasal	White	33/F	Pericardial tamponade	Died without chemotherapy	Died
10. 9. Front Cardiovasc Med.2021. 8: 685736.	Nasal	Yellow	40/F	Myocarditis	Died without chemotherapy	Died
11. 10. Case Rep Hematol. 2016.2016: 2394809.	Non-nasal	White	62/M	Arrhythmia	Died without chemotherapy	Died
11.Leuk Lymphoa.2008. 49(5): 1008-11.	Non-nasal	Yellow	42/M	Arrhythmia	Death due to cardiac arrhythmia during chemotherapy	Died
12. Ocul Oncol Pathol. 2018. 4(6): 388-394.	Non-nasal	Black	53/M	Pericardial tamponade	Death during chemotherapy	Died
13.Hum Pathol.2003.34(3):290-2.	Non-nasal	Yellow	81/M	Myocardil hypertrophy	Died without chemotherapy	Died

These features were also seen in our reported cases. However, unlike the previously reported cases, our case has its own characteristics: history of hypertrophic obstructive cardiomyopathy. Because of this feature, the patient’s echocardiographic findings were not emphasized in the early stages. At the same time, because the history of heart disease was longer than the time of its nodal onset, it was thought to be a recurrence of hypertrophic obstructive cardiomyopathy triggering cardiac problems. These mislead clinicians about the diagnosis of the disease. In addition, to the best of our knowledge, this is the first case of ENKTL invading the heart in a patient with hypertrophic obstructive cardiomyopathy.

As ENKTL is one of the most difficult subtypes to treat and is associated with a poor prognosis, current treatment strategies have not been clear defined. Based on the only two surviving cases with successful chemotherapy, after successful chemotherapy for ENKTL invasion of the heart, significant improvements in various parameters such as echocardiography were observed, indicating that the results it caused could be reversed by chemotherapy (7, 8). Therefore, despite the adverse effects such as malignant arrhythmias that can occur in patients during chemotherapy, chemotherapy is still recommended as the preferred method once the diagnosis is confirmed. For the present, there is no standard chemotherapy regimen for ENKTL. In our case, we used the P-Gemox chemotherapy regimen, which has entered the National Comprehensive Cancer Network (NCCN) guidelines. In an oral presentation at ASH, the combination of Sintilimab and Chidamide was shown to have good antitumor activity in the treatment of patients with refractory or relapsed nasal ENKTL. In 36 refractory patients with evaluable efficacy, an overall efficacy rate of 58.3%, a complete remission rate of 44.4%, and an effective maintenance time of more than 9.2 months were achieved with a manageable safety profile (15). In contrast, the SMILE regimen used in successful cases has side effects that are so strong that patients may not tolerate them. Moreover, in a Japanese multicenter study, the SMILE regimen was reported to be ineffective in non-nasal type patients, with a 2-year overall survival of 34%, while it was more effective in nasal type patients, with a 2-year overall survival of 70% (16). Hence, the regimen we have adopted is already at the forefront of international regimens and is appropriate for the patient’s condition. Despite this, the patient’s skin nodules and heart failure continued to progress under this chemotherapy regimen. This makes us wonder about the refractory nature of ENKTL. Current considerations for the refractoriness and recurrence of ENKTL are due to immune evasion, some studies have shown that ENKTL cells avoid immune surveillance and the consequent killing of ENKTL, resulting in a poor outcome (17). This may be the reason why patients are resistant to Sintilimab. Such an outcome corresponds exactly to the poor prognosis of non-nasal ENKTL. This calls for further exploration of regimens for this disease.

Our case together with the summary of previous case reports illustrate (1). Non-nasal ENKTL is more likely to invade the heart and has a poorer prognosis than the nasal type (2). Our case is the first case of ENKTL invading the heart in a patient with hypertrophic obstructive cardiomyopathy (3). The regimen we used is already extremely cutting-edge clinically and has better efficacy and longer survival time for refractory recurrent ENKTL compared to the traditional SMILE regimen. Nonetheless, the results are still poor for our patients, which warrants for further exploration of chemotherapy regimens for this type of disease.

## Data availability statement

The raw data supporting the conclusions of this article will be made available by the authors, without undue reservation.

## Ethics statement

The studies involving human participants were reviewed and approved by Fujian Provincial Hospital ethics committee. Written informed consent for participation was not required for this study in accordance with the national legislation and the institutional requirements.

## Author contributions

YX was responsible for the clinical design and conceptualization. YX and XL wrote the manuscript. XC was involved in the acquisition of the clinical data. SQ and K-YL conducted the clinical diagnosis. All authors discussed, read, and approved the submission of this manuscript to the journal.
